# Supplementary material for: The Ragulator complex and lysosomal calcium release are crucial for cell migration
Source: Life Sci Alliance. 2025 Jun 10;8(8):e202403015. doi: 10.26508/lsa.202403015 (PMC12152492; doi:10.26508/lsa.202403015)

1A. Effect of calcium on interaction between Lamtor1 and MPRIP

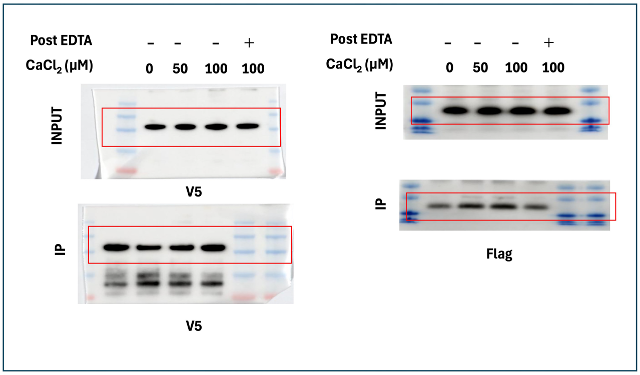

1C. The immunoprecipitation assay between TRPML1 and Lamtor1

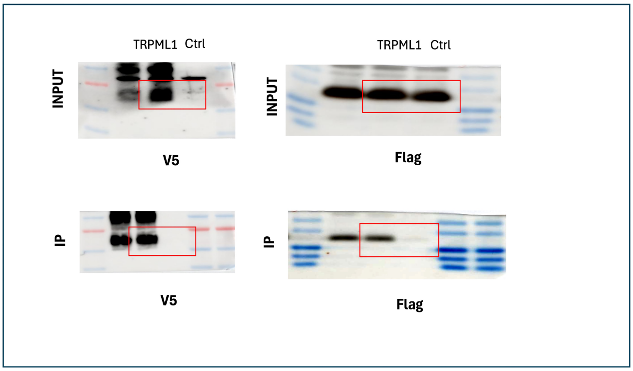

1D. Effect of MLSA-1 on interaction between Lamtor1 and MPRIP

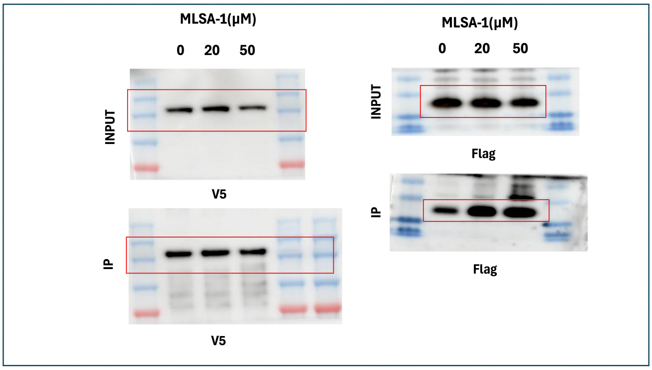

1G. Effects of MLSA-1 on MLC phosphorylation

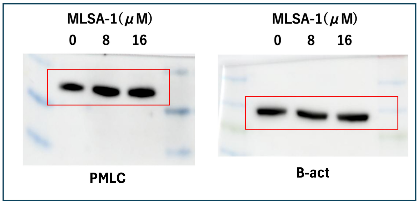

Effects of MLSI-3 on MLC phosphorylation

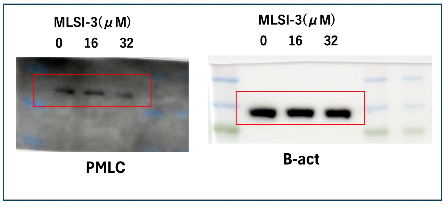

Supplement: Supplementary file 1 [file LSA-2024-03015_SdataF1.1.pdf]
