# Supplementary material for: The Ragulator complex and lysosomal calcium release are crucial for cell migration
Source: Life Sci Alliance. 2025 Jun 10;8(8):e202403015. doi: 10.26508/lsa.202403015 (PMC12152492; doi:10.26508/lsa.202403015)

S1A. Effect of A23187 on the interaction between Lamtor1 and MPRIP

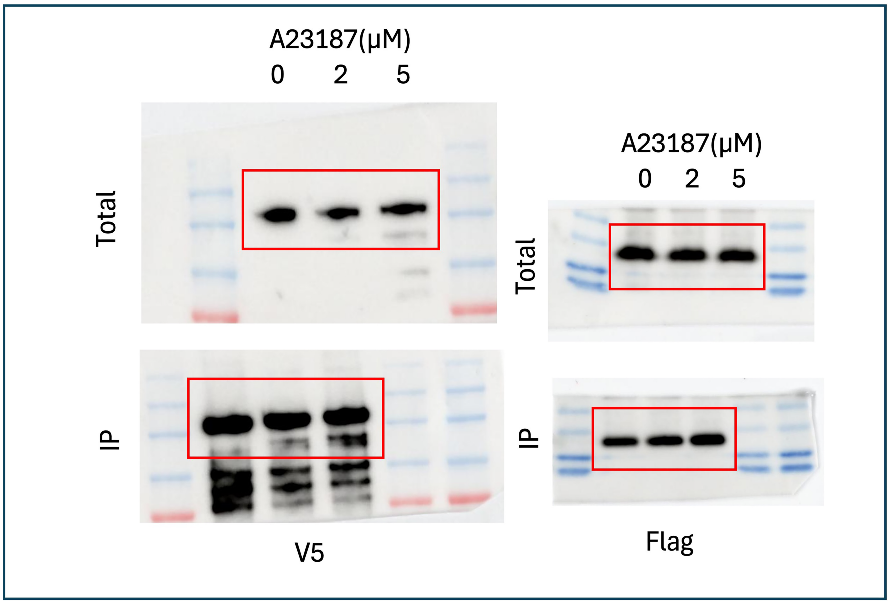

S1C. Effect of MLSI-3 on lysosome distribution during DC migration.

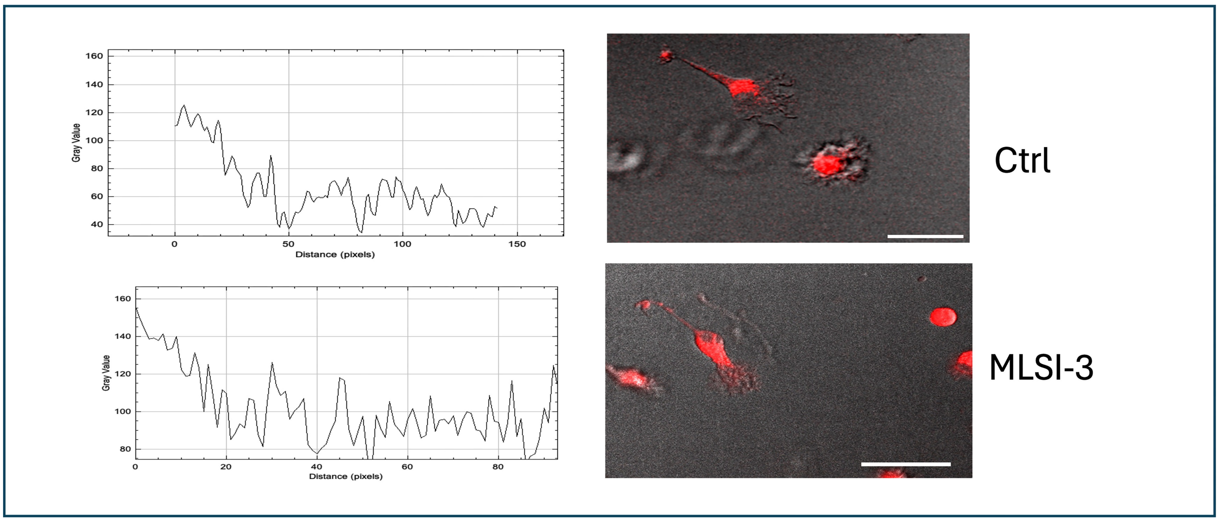

Supplement: Supplementary file 4 [file LSA-2024-03015_SdataFS1.2.pdf]
