# Supplementary figures and images for: The Ragulator complex and lysosomal calcium release are crucial for cell migration
Source: Life Sci Alliance. 2025 Jun 10;8(8):e202403015. doi: 10.26508/lsa.202403015 (PMC12152492; doi:10.26508/lsa.202403015)

S2B. Lysosome fractions of Lamtor1-G2A in THP-1 cells.

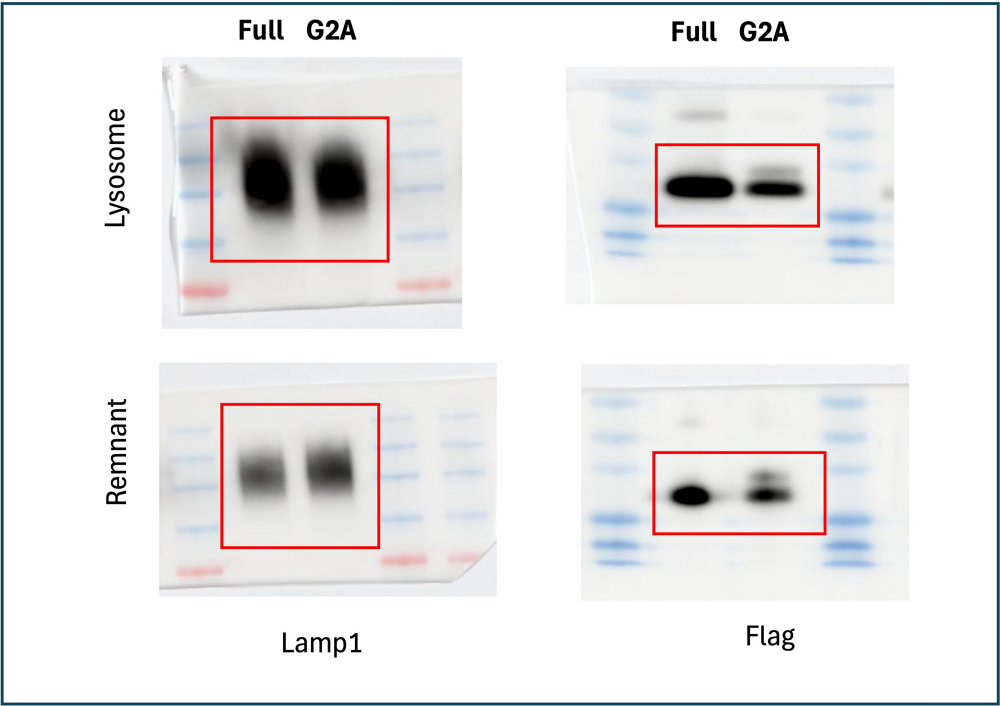

Supplement: Supplementary file 12 [file LSA-2024-03015_SdataFS2.1.pdf]
