# Supplementary material for: The Ragulator complex and lysosomal calcium release are crucial for cell migration
Source: Life Sci Alliance. 2025 Jun 10;8(8):e202403015. doi: 10.26508/lsa.202403015 (PMC12152492; doi:10.26508/lsa.202403015)

Figure 2A. Effect of disrupting lysosomal localization of Lamtor1 on MPRIP interactions

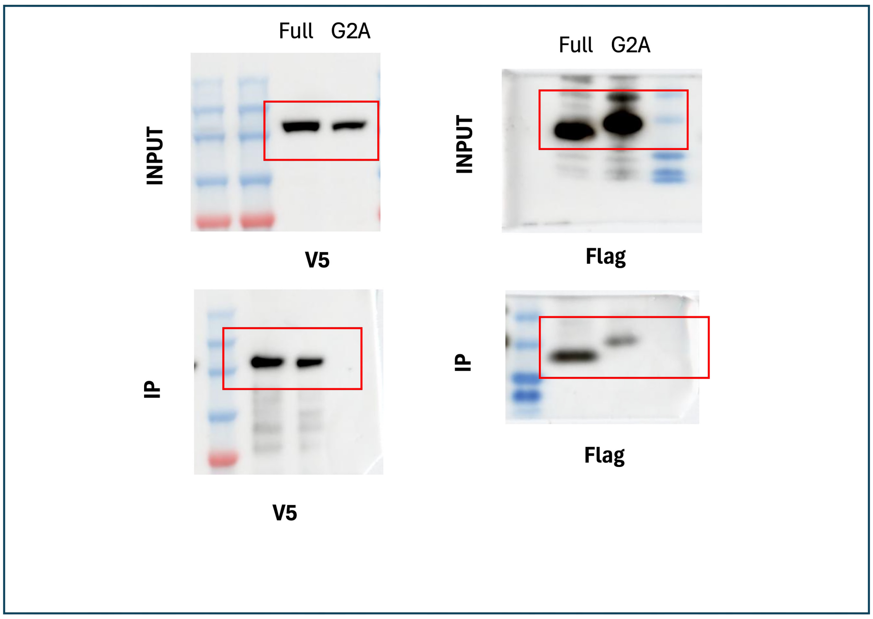

Figure 2C. Effect of Lamtor1 G2A on MLC phosphorylation

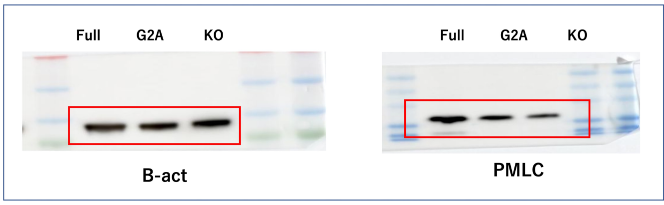

Figure 2D. Effects of MLSA-1 on the interaction between Lamtor1 G2A and MPRIP

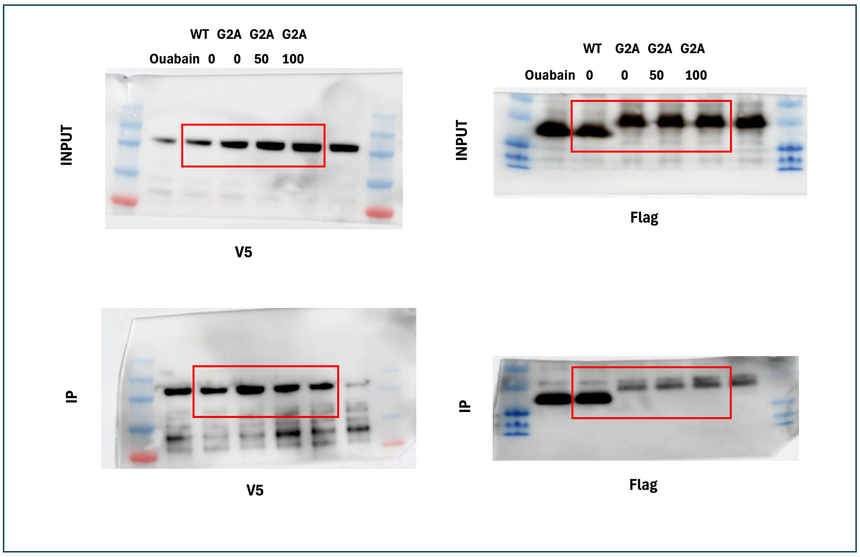

Figure 2F. Effect of MLSA-1 on MLC phosphorylation of Lamtor1-G2A.

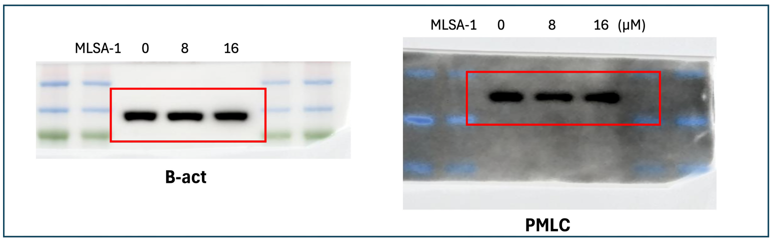

Supplement: Supplementary file 14 [file LSA-2024-03015_SdataF2.1.pdf]
