# Supplementary material for: The Ragulator complex and lysosomal calcium release are crucial for cell migration
Source: Life Sci Alliance. 2025 Jun 10;8(8):e202403015. doi: 10.26508/lsa.202403015 (PMC12152492; doi:10.26508/lsa.202403015)

3B. Effect of ouabain on the interaction between Lamtor1-MPRIP interaction

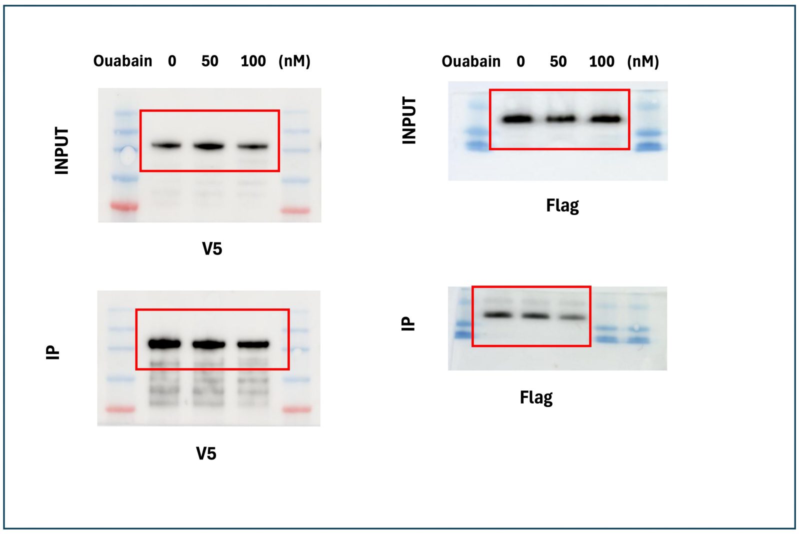

3C. Effect of ouabain on MYPT1-MPRIP interaction

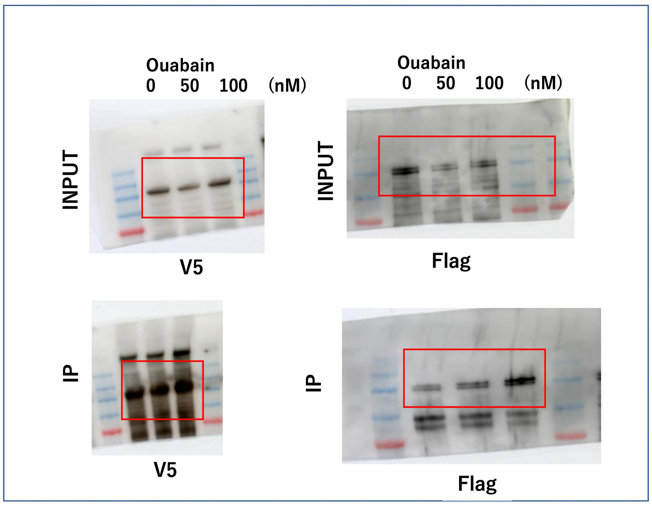

3E. Localization of Lamtor1 after ouabain treatment

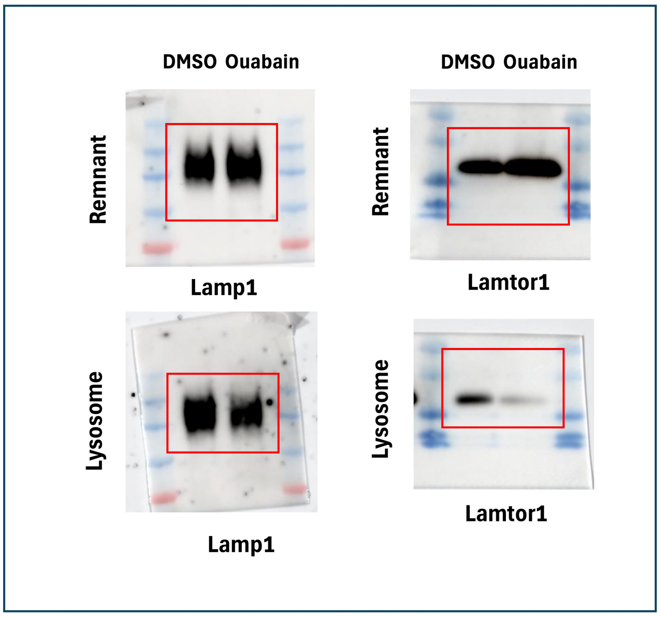

3F. Attenuation of MLSA-1 mediated Lamtor1-MPRIP interaction by ouabain

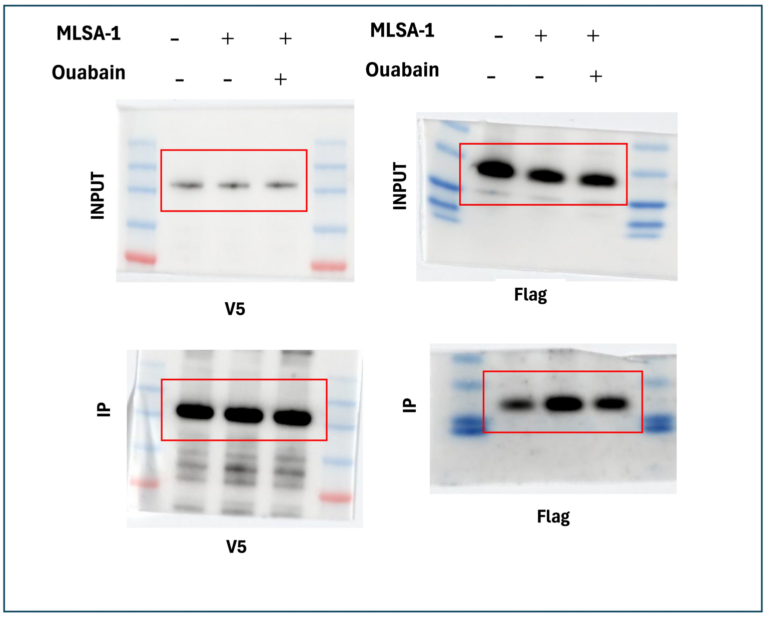

Supplement: Supplementary file 16 [file LSA-2024-03015_SdataF3.1.pdf]
