# Supplementary material for: The Ragulator complex and lysosomal calcium release are crucial for cell migration
Source: Life Sci Alliance. 2025 Jun 10;8(8):e202403015. doi: 10.26508/lsa.202403015 (PMC12152492; doi:10.26508/lsa.202403015)

S3A. Interaction between Lamtor1-5 and MPRIP

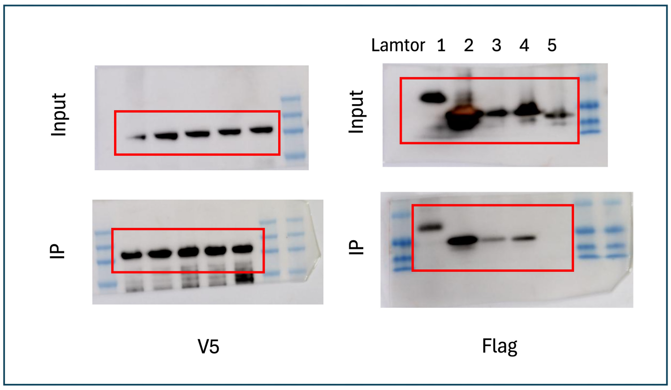

S3B. Interaction between Lamtor1 and MPRIP truncate form

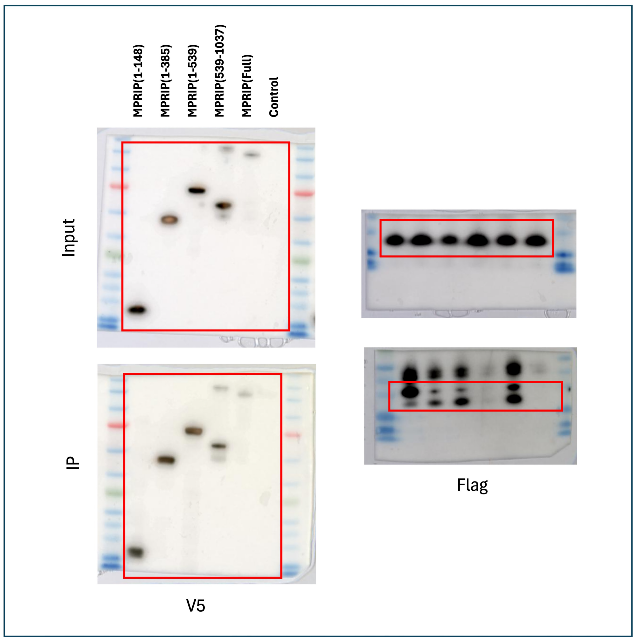

S3D. The effects of ouabain on mTORC1 activity

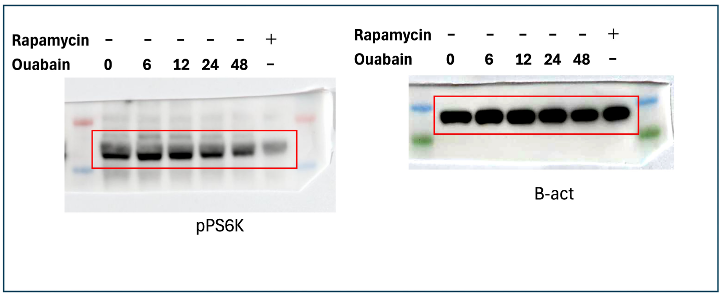

S3E. The effects of ouabain on LC3 efflux

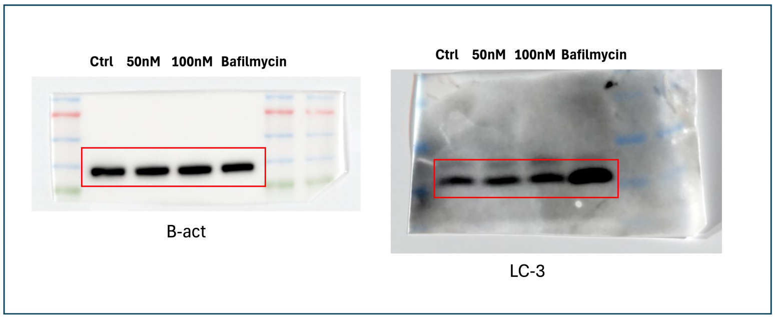

S3G. The interaction of Lamtor1-Flag with Lamtor2-5 in the Lamtor1-G2A mutant or under ouabain treatment.

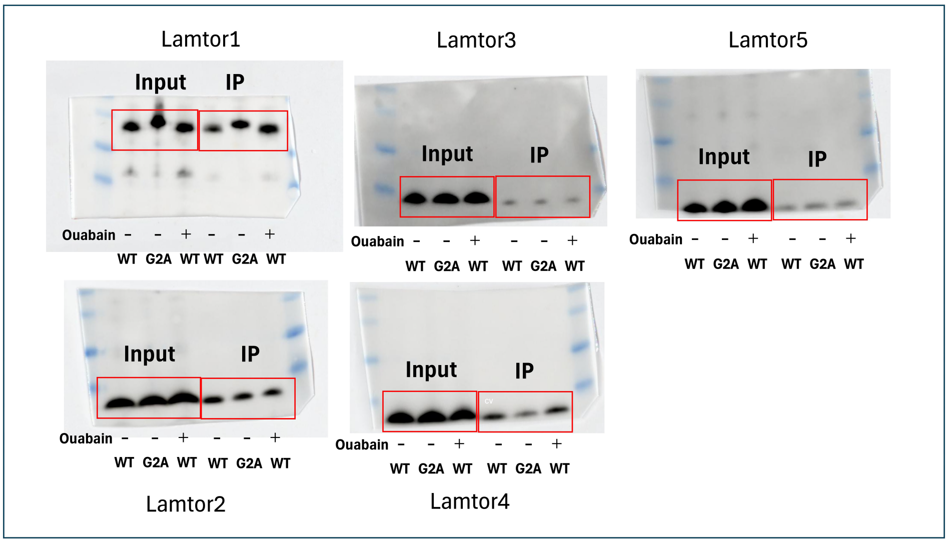

S3H. Western blotting showing ATP1A1 KD

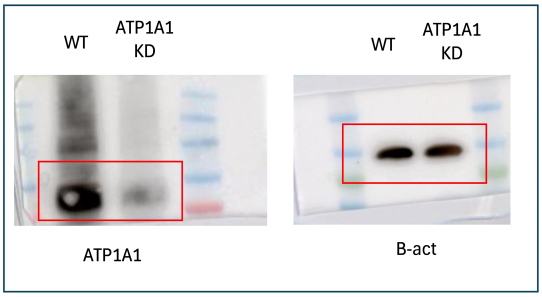

Supplement: Supplementary file 19 [file LSA-2024-03015_SdataFS3.1.pdf]
