# Supplementary material for: The Ragulator complex and lysosomal calcium release are crucial for cell migration
Source: Life Sci Alliance. 2025 Jun 10;8(8):e202403015. doi: 10.26508/lsa.202403015 (PMC12152492; doi:10.26508/lsa.202403015)

4B. Effects of ouabain on MLC phosphorylation.

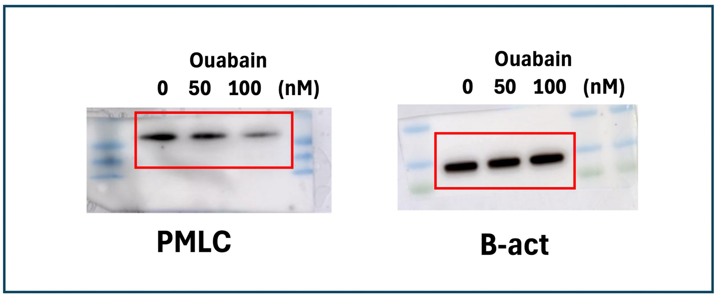

4C. Rac1 expression was assessed using the Active Rac1 Detection Kit

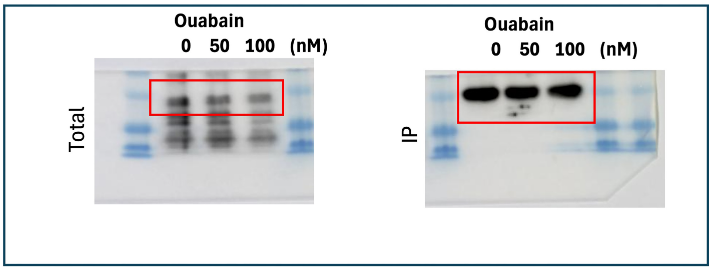

4C. GTP-RhoA levels were assessed using Active RhoA Detection Kit

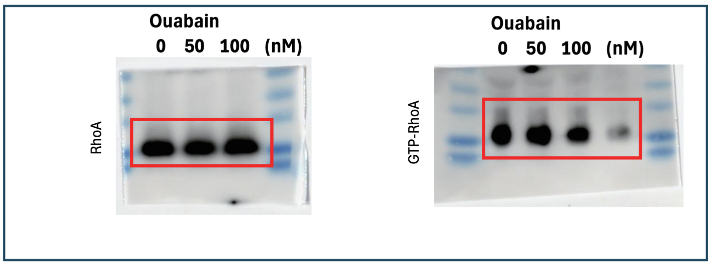

4E. Effect of ouabain in an acute gouty arthritis model

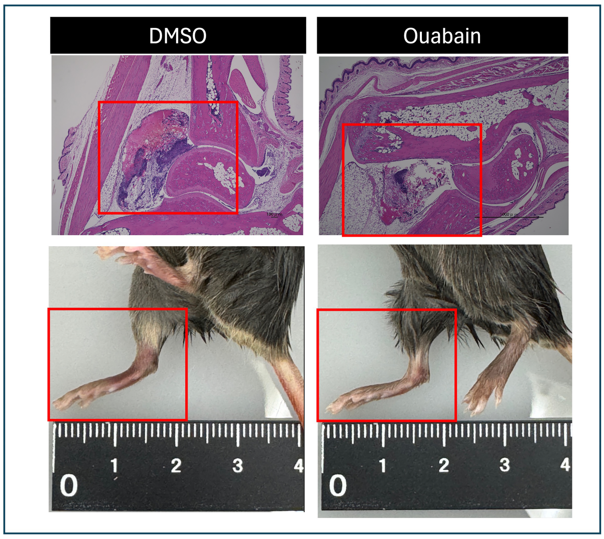

4F. Effect of ouabain in the LPS-induced acute lung injury model

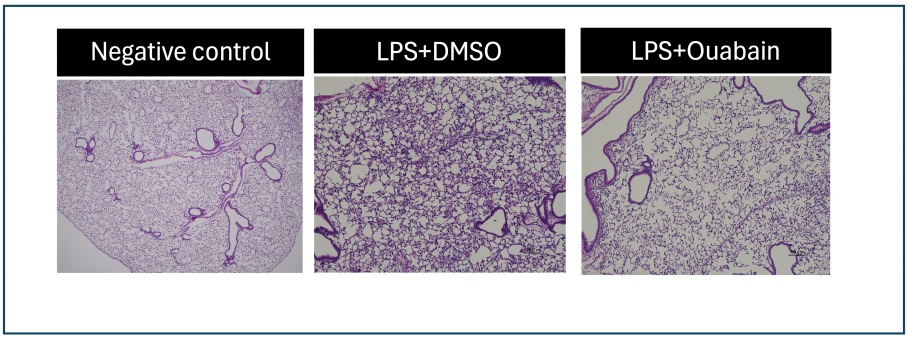

4G. Effects of ouabain on neutrophil recruitment in an Alum-induced peritonitis model.

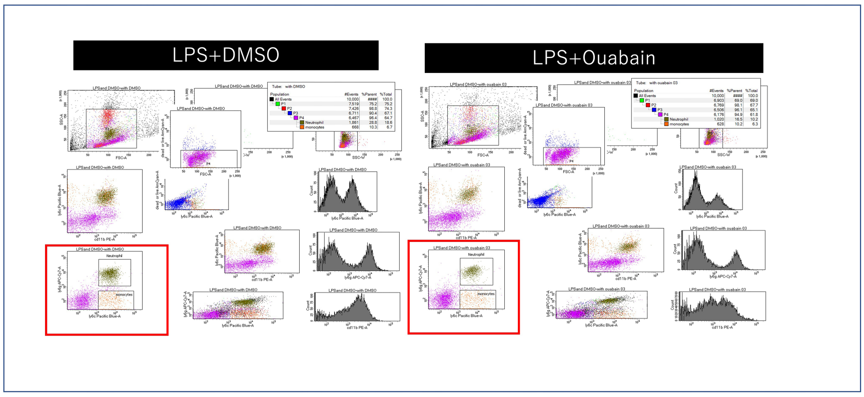

Supplement: Supplementary file 21 [file LSA-2024-03015_SdataF4.1.pdf]
