# Supplementary material for: The Ragulator complex and lysosomal calcium release are crucial for cell migration
Source: Life Sci Alliance. 2025 Jun 10;8(8):e202403015. doi: 10.26508/lsa.202403015 (PMC12152492; doi:10.26508/lsa.202403015)

S4A. Western blot showing MPRIP-KO

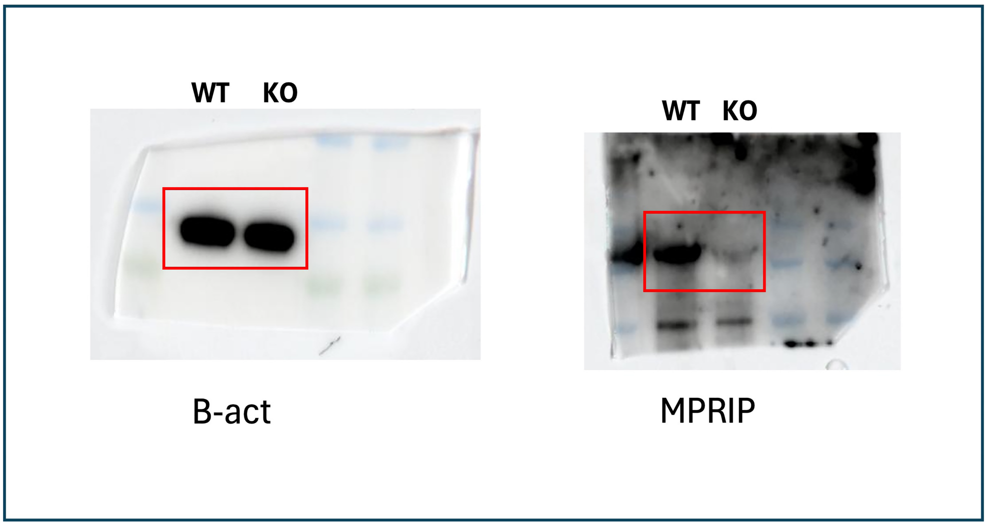

S4C. Effects of ouabain on MLC phosphorylation in Lamtor1-KO-THP1

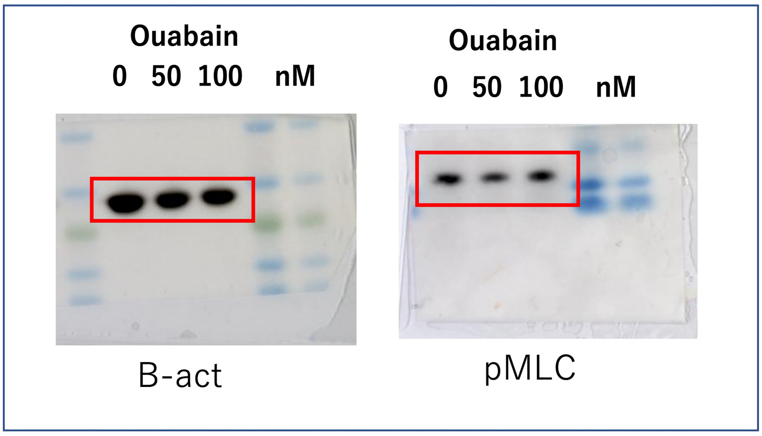

S4C. Effects of ouabain on MLC phosphorylation in MPRIP-KO-THP1 cells

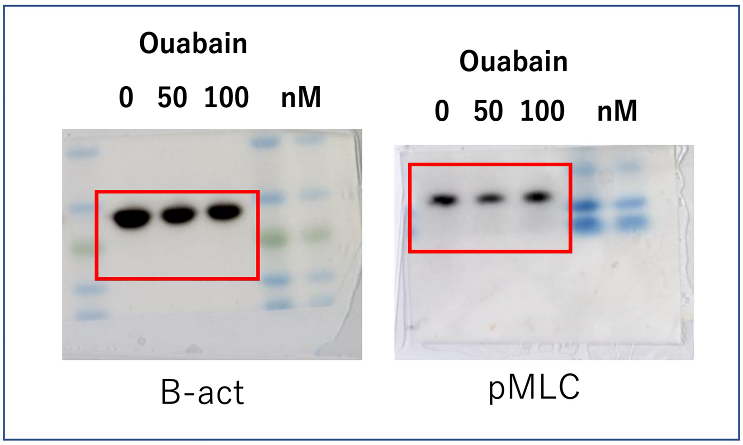

Supplement: Supplementary file 23 [file LSA-2024-03015_SdataFS4.1.pdf]
