# Supplementary material for: The Ragulator complex and lysosomal calcium release are crucial for cell migration
Source: Life Sci Alliance. 2025 Jun 10;8(8):e202403015. doi: 10.26508/lsa.202403015 (PMC12152492; doi:10.26508/lsa.202403015)
Supplement: Supplementary file 25 [file LSA-2024-03015_TableS1.docx]

Reagents and Table

**The Ragulator complex and lysosomal calcium release**

**are crucial for cell migration**

Tatsunori Jo,

Kohei Tsujimoto, Takeshi Nakatani, Daiki Nagira,

Yutaka Muto, Takehiro Hirayama, Hachiro Konaka, Masato Okada,

Hyota Takamatsu, and Atsushi Kumanogoh.

Tables and legends.

| **Antibodies**  **(Western blot and Immunoprecipitation)** | | |
| --- | --- | --- |
|  | **Vendor** | **Catalog number** |
| Phospho-Myosin Light Chain 2(Ser19) Antibody | Cell Signaling Technology | #3671 |
| Phospho-p70 S6 Kinase (Thr389) Rabbit mAb | Cell Signaling Technology | #2708 |
| β-Actin Rabbit mAb (HRP Conjugate) | Cell Signaling Technology | #5125 |
| M-RIP Rabbit mAb | Sigma | HPA022901 |
| DYKDDDDK Tag Antibody (immunoprecipitation) | Cell Signaling Technology | 2368 |
| Anti-V5 Antibody (immunoprecipitation) | Invitrogen | 46-0705 |
| Monoclonal ANTI-FLAG® M2-Peroxidase (HRP) antibody (Western) | Sigma | A-8592 |
| LAMTOR1/C11orf59 XP® Rabbit mAb | Cell Signaling Technology | #8975 |
| LAMTOR2/ROBLD3 Rabbit mAb | Cell Signaling Technology | #8145 |
| LAMTOR3/MAPKSP1 Rabbit mAb | Cell Signaling Technology | #8168 |
| LAMTOR4/C7orf59 Rabbit mAb | Cell Signaling Technology | #12284 |
| LAMTOR5/HBXIP Rabbit mAb | Cell Signaling Technology | #14633 |
| Myc-tag (9B11) | Cell Signaling Technology | 2276S |
| Sodium Potassium ATPase Recombinant Rabbit Monoclonal Antibody | Invitrogen | MA5-32184 |
| V5 Tag Monoclonal Antibody, HRP | Invitrogen | 46-0708 |
| RhoA (67B9) Rabbit mAb | Cell Signaling Technology | #2117 |
| Rac1/Cdc42 Antibody | Cell Signaling Technology | #4651 |
| LC3B (D11) XP® Rabbit mAb | Cell Signaling Technology | #3868 |

| **Antibodies for Immunohistochemistry** | | |
| --- | --- | --- |
|  | **Vendor** | **Catalog number** |
| F(ab')2-Goat anti-Mouse IgG (H+L) Cross-Adsorbed Secondary Antibody, Alexa Fluor™ 488 | Invitrogen | A-11017 |
| Goat anti-Rabbit IgG (H+L) Cross-Adsorbed Secondary Antibody, Alexa Fluor™ 647 | Invitrogen | A-21244 |
| ANTI-FLAG® M2-Peroxidase (HRP) mAb | Sigma | F3165 |
| MCOLN1 Polyclonal antibody | proteintech | 15291-1-AP |
| Anti-LAMP1 antibody [EPR21026] | Abcam | ab208943 |
| Anti-LAMP1 antibody [H4A3] | Abcam | ab25630 |
| LAMTOR1/C11orf59 XP® Rabbit mAb | Cell Signaling Technology | #8975 |

| **Antibodies for flow cytometry** | | |
| --- | --- | --- |
|  | **Vendor** | **Catalog number** |
| PE anti-mouse/human CD11b Antibody | Biolegend | 101207 |
| APC/Cyanine7 anti-mouse Ly-6G Antibody | Biolegend | 127623 |
| Brilliant Violet 421™ anti-mouse Ly-6C Antibody | Biolegend | 128031 |
| PerCP/Cyanine5.5 Annexin V | Biolegend | 640935 |
| FITC anti-mouse CD80 Antibody | Biolegend | 104705 |
| PE anti-mouse CD86 Antibody | Biolegend | 159203 |

| **Reagent** | | |
| --- | --- | --- |
|  | **Vendor** | **Catalog number** |
| LPS | Sigma-Aldrich | L3024 |
| PMA | Sigma-Aldrich | P8139-1MG |
| CFSE | Thermo Fisher | C34554 |
| Calcein AM | Thermo Fisher | C1430 |
| Recombinant human MCP-1 | R & D | 279-MC-010 |
| Mouse GM-CSF | Wako | 077-04674 |
| MISSION shRNA plasmid for ATP1A1 | Sigma-Aldrich | TRCN0000043226 |
| MSU | InvivoGen | tlrl-msu |
| Torin | Selleck | S2827 |
| 1-O-n-Octyl-β-D-glucopyranoside | Nacalai Tesque | 25535-24 |
| Lipofectamine 2000 | Thermo Fisher | 11668-019 |
| Imject Alum | Thermo Fisher | 77161 |
| MLSA-1 | Selleck | S9926-25MG |
| MLSI-3 | Selleck | E0026 |
| Ouabain | Selleck | S4016 |
| A23187 | Sigma-Aldrich | C7522 |
| Calcein AM |  |  |

]

| **Primers for expression vector generation** |
| --- |
| Lamtor1-NanoLuc  Fw: GATCGCTTCCGAATTATGGGGTGCTGCTATAGCAGC  Rv: GCCGCTCGAGCCGAGCTTGTCATCGTCATCCTTGTAGTC |
| Halotaq-MPRIP  Fw: GATCGCTTCCGAATTATGGGGTGCTGCTATAGCAGC  Rv: GCCGCTCGAGCCGAGCTTGTCATCGTCATCCTTGTAGTC |
| Lamtor2-NanoLuc  Fw: GATCGCTTCCGAATTTATGTCGGCGGCCAAGGAAAAC  Rv: GCCGCTCGAGCCGAGCTTGTCATCGTCATCCTTGTAATCG |
| MPRIP 1-150  Fw: AATTCTGCAGCGGCCATGTCGGCGGCCAAGGAAAAC  Rv:GGAGAGGGGCGGATCCGTAGAATCGAGACCGAGGAGAGGGTTAGGGATAGGCTTACCTCGAGGATACACCATGAGCATCTC |
| MRIP 1-384  Fw: AATTCTGCAGCGGCCATGTCGGCGGCCAAGGAAAAC  Rv:GGAGAGGGGCGGATCCGTAGAATCGAGACCAGGAGAGGGTTAGGGATAGGCT  TACCAGTCAGGCGTCATGGAGGATTCTGT |
| MPRIP 1-539  Fw: AATTCTGCAGCGGCCATGTCGGCGGCCAAGGAAAAC  Rv: GGAGAGGGGCGGATCCGTAGAATCGAGACCGAGGAGAGG |
| MPRIP 539-1037  Fw: AATTCTGCAGCGGCCATGTCGGCGGCCAAGGAAAAC  Rv: GGAGAGGGGCGGATCATCTTTCTTCAAGTCCCTGGATTCA |

| **Primers for KO cell line generation** |
| --- |
| **Generation of MPRIP-KO-THP1**   1. 5’CGTCGCTGCCATTTCTGCAACGG3’ 2. 5’TCCGGATATGGTGCGTCCTCGGG3’ 3. 5’ACCCGAGGACGCACCATATCCGG3’ 4. 5’CGCACCATATCCGGACACCGTGG3’ |
| **Generation of ATP1A1-KD THP1**  CCGGCCTGCTGACCTCAGAATCATACTCGAGTATGATTCTGA  GGTCAGCAGGTTTTTG |

| **Gene Synthesis** |
| --- |
| **Generation of TRPML1-V5 （Eurofin　Genomics）**  ATGACAGCCCCGGCGGGTCCGCGCGGCTCAGAGACCGAGCGGCTTCTGACCCCCAACCCCGGGTATGGGACCCAGGCGGGGCCTTCACCGGCCCCTCCGACACCCCCAGAAGAGGAAGACCTTCGCCGTCGTCTCAAATACTTTTTCATGAGTCCCTGCGACAAGTTTCGAGCCAAGGGCCGCAAGCCCTGCAAGCTGATGCTGCAAGTGGTCAAGATCCTGGTGGTCACGGTGCAGCTCATCCTGTTTGGGCTCAGTAATCAGCTGGCTGTGACATTCCGGGAAGAGAACACCATCGCCTTCCGACACCTCTTCCTGCTGGGCTACTCGGACGGAGCGGATGACACCTTCGCAGCCTACACGCGGGAGCAGCTGTACCAGGCCATCTTCCATGCTGTGGACCAGTACCTGGCGTTGCCTGACGTGTCACTGGGCCGGTATGCGTATGTCCGTGGTGGGGGTGACCCTTGGACCAATGGCTCAGGGCTTGCTCTCTGCCAGCGGTACTACCACCGAGGCCACGTGGACCCGGCCAACGACACATTTGACATTGATCCGATGGTGGTTACTGACTGCATCCAGGTGGATCCCCCCGAGCGGCCCCCTCCGCCCCCCAGCGACGATCTCACCCTCTTGGAAAGCAGCTCCAGTTACAAGAACCTCACGCTCAAATTCCACAAGCTGGTCAATGTCACCATCCACTTCCGGCTGAAGACCATTAACCTCCAGAGCCTCATCAATAATGAGATCCCGGACTGCTATACCTTCAGCGTCCTGATCACGTTTGACAACAAAGCACACAGTGGGCGGATCCCCATCAGCCTGGAGACCCAGGCCCACATCCAGGAGTGTAAGCACCCCAGTGTCTTCCAGCACGGAGACAACAGCTTCCGGCTCCTGTTTGACGTGGTGGTCATCCTCACCTGCTCCCTGTCCTTCCTCCTCTGCGCCCGCTCACTCCTTCGAGGCTTCCTGCTGCAGAACGAGTTTGTGGGGTTCATGTGGCGGCAGCGGGGACGGGTCATCAGCCTGTGGGAGCGGCTGGAATTTGTCAATGGCTGGTACATCCTGCTCGTCACCAGCGATGTGCTCACCATCTCGGGCACCATCATGAAGATCGGCATCGAGGCCAAGAACTTGGCGAGCTACGACGTCTGCAGCATCCTCCTGGGCACCTCGACGCTGCTGGTGTGGGTGGGCGTGATCCGCTACCTGACCTTCTTCCACAACTACAATATCCTCATCGCCACACTGCGGGTGGCCCTGCCCAGCGTCATGCGCTTCTGCTGCTGCGTGGCTGTCATCTACCTGGGCTACTGCTTCTGTGGCTGGATCGTGCTGGGGCCCTATCATGTGAAGTTCCGCTCACTCTCCATGGTGTCTGAGTGCCTGTTCTCGCTCATCAATGGGGACGACATGTTTGTGACGTTCGCCGCCATGCAGGCGCAGCAGGGCCGCAGCAGCCTGGTGTGGCTCTTCTCCCAGCTCTACCTTTACTCCTTCATCAGCCTCTTCATCTACATGGTGCTCAGCCTCTTCATCGCGCTCATCACCGGCGCCTACGACACCATCAAGCATCCCGGCGGCGCAGGCGCAGAGGAGAGCGAGCTGCAGGCCTACATCGCACAGTGCCAGGACAGCCCCACCTCCGGCAAGTTCCGCCGCGGGAGCGGCTCGGCCTGCAGCCTTCTCTGCTGCTGCGGAAGGGACCCCTCGGAGGAGCATTCGCTGCTGGTGAATTGTGGTAAGCCTATCCCTAACCCTCTCCTCGGTCTCGATTCTACGTAGTGA |
